# Supplementary material for: Photonic crystal enhanced fluorescence emission and blinking suppression for single quantum dot digital resolution biosensing
Source: Nat Commun. 2022 Aug 8;13:4647. doi: 10.1038/s41467-022-32387-w (PMC9360002; doi:10.1038/s41467-022-32387-w)
Supplement: Supplementary file 1 — Supplementary Information [file 41467_2022_32387_MOESM1_ESM.pdf]

Supplementary Information

Photonic Crystal Enhanced Fluorescence Emission and Blinking Suppression for Single Quantum Dot Digital Resolution Biosensing

Authors:

Yanyu Xiong[1,2], Qinglan Huang[1,2], Taylor D. Canady[2,3], Priyash Barya[1,2], Shengyan Liu[1,2], Opeyemi H. Arogundade[4], Caitlin M. Race[1,2], Congnyu Che[2,4], Xiaojing Wang[2,3], Lifeng Zhou[2,3], Xing Wang[2,3,4], Manish Kohli[5], Andrew M. Smith[2,4,6,7,8], and Brian T. Cunningham[1,2,3,4,8*]

Affiliations:

[1] Department of Electrical and Computer Engineering, University of Illinois at Urbana−Champaign, Urbana, Illinois, 61801, USA

[2] Holonyak Micro and Nanotechnology Laboratory, University of Illinois at Urbana−Champaign, Urbana, Illinois, 61801, USA

[3] Carl R. Woese Institute for Genomic Biology, University of Illinois at Urbana−Champaign, Urbana, Illinois, 61801, USA

[4] Department of Bioengineering, University of Illinois at Urbana-Champaign, Urbana, Illinois, 61801, USA

[5] Department of Oncology, Huntsman Cancer Institute, Salt Lake City, UT, 84112, USA

[6] Carle Illinois College of Medicine, Urbana, Illinois, 61801, USA

[7] Department of Materials Science and Engineering, University of Illinois at Urbana-Champaign, Urbana, Illinois, 61801, USA

[8] Cancer Center at Illinois, Urbana, IL, 61801, USA

* To whom correspondence may be addressed. Email: bcunning@illinois.edu

1. Design and Characterization of the Photonic Crystal (PC)

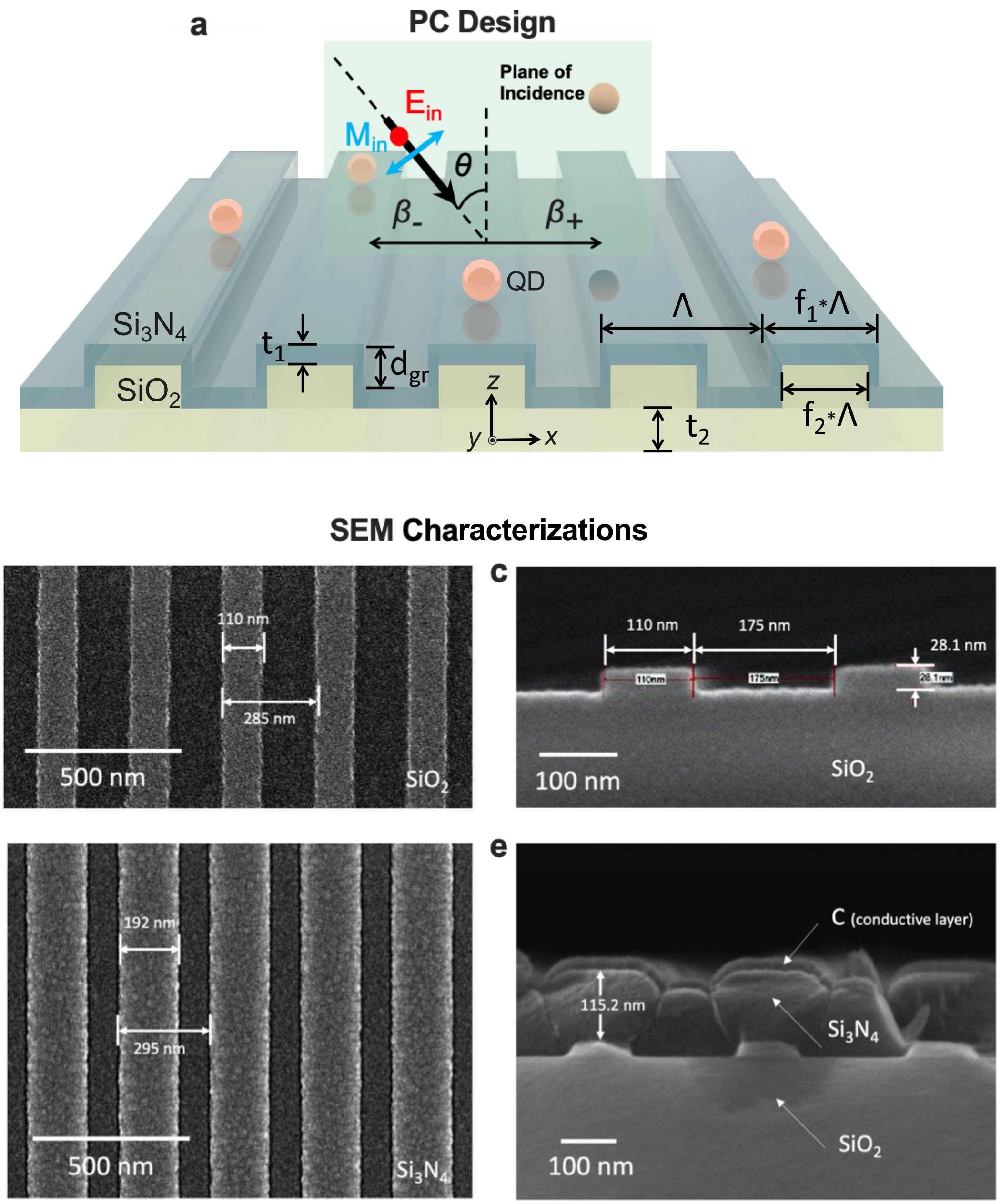

Supplementary Figure S1. Design and Characterization of the Photonic Crystal (PC) Structure. (a). QDs are spread onto the PC surface. A 5 nm thick TiO_2 coating applied over the PC top surface is omitted in the schematic. The excitation laser (λ_{laser} = 450 nm) is TE-polarized with an incidence angle θ. Structure parameters of the PC: Λ = 295 nm, d_{gr} = 28.1 nm, t_1 = 115

nm, t_2 = 1µm, f_1 = 0.651, f_2 = 0.385; The Bird's-eye view (b) and cross-section view (c) of SEM characterizations of PC SiO_2 periodic structure before Si_3N_4 deposition. The Bird's-eye view (d) and cross-section view (e) of SEM characterizations of PC Si_3N_4 top-grating structure. Since the partial fracture and separation during the cleaving process obscured the underlying SiO_2 rib dimensions, we use the SEM image before Si_3N_4 deposition to determine the SiO_2 structure dimensions. More than three experiments were repeated independently with similar results.

2. Far-field Reflection Spectrum of the PC pump-mode

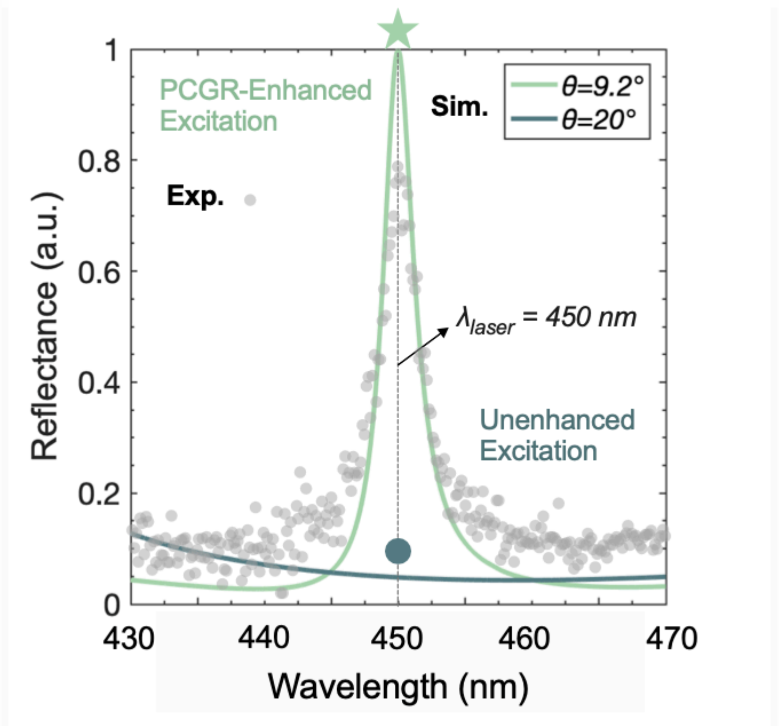

Supplementary Figure S2. Far-field Reflection Spectrum of the PC pump-mode. Simulated (lines) and experimental measured (points) far-field reflectance spectrum of the PC, when it is on-resonance for the PCGR enhanced pump-mode (θ = 9.2°, light green star) or off-resonance (θ = 20°, dark green dot).

3. Photonic Crystal Enhanced Fluorescence (PCEF) Optical Setup

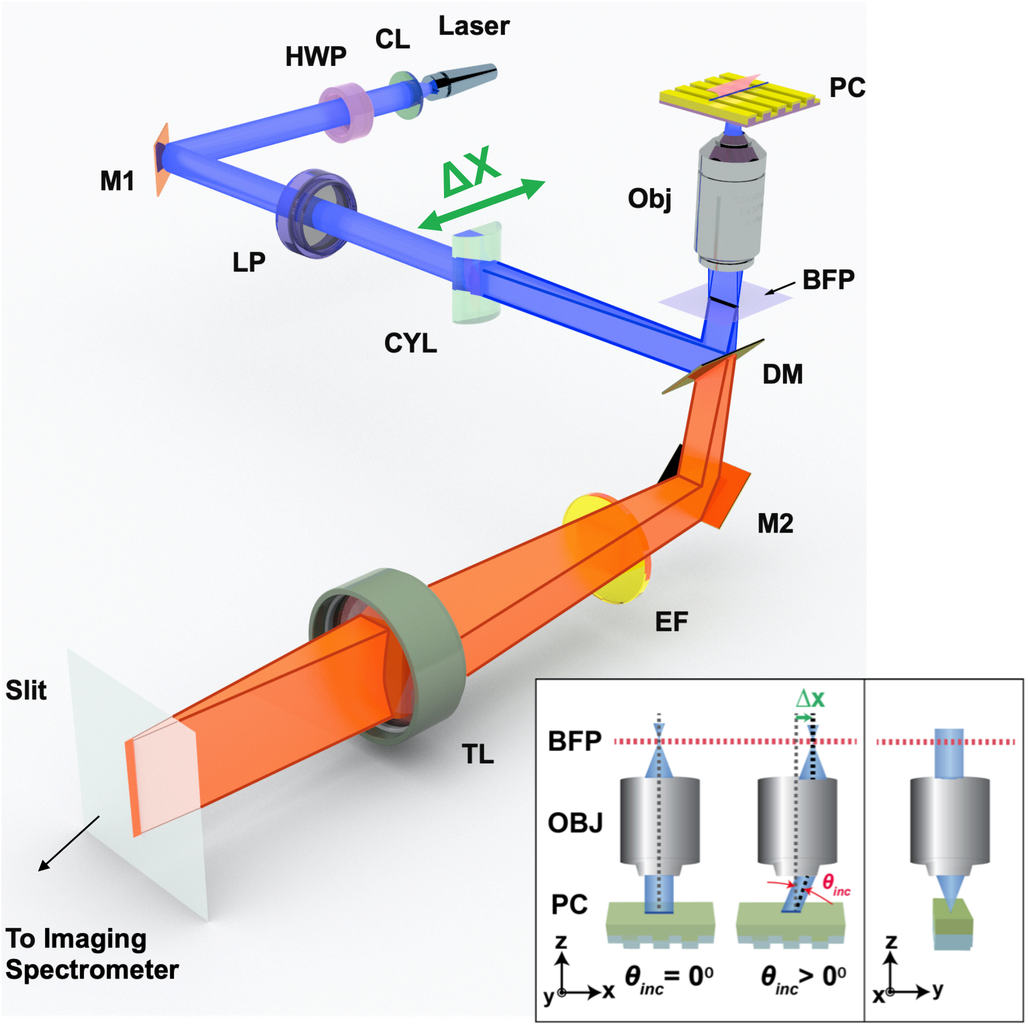

Supplementary Figure S3. Photonic Crystal Enhanced Fluorescence (PCEF) Optical Setup. Laser: laser diode (450 nm), CL: collimating lens, HWP: half-wave plate, LP: linear polarizer, CYL: cylindrical lens, DM: dramatic Mirror, OBJ: objective Lens, TL: tube lens, M1, M2: Mirrors, EF: emission filter (500 nm long-pass), BFP: back focal plane.

4. Enhancement excitation and extraction factor simulation.

$$\Lambda_{excitation} \equiv \frac{P_{\mathrm{PC}}^{abs}}{P_{\mathrm{Glass}}^{abs}} = \frac{2\lambda_{laser}}{\pi n d} \frac{\alpha^{P} \left(Q^{P}\right)^{2}}{Q_{\mathrm{r}}^{P}} \tag{1}$$

$$\Lambda_{extraxtion}\left(\mathbf{k}, \omega_{\mathbf{k}}\right) \equiv \frac{\Gamma^{\mathrm{PC}} \times \frac{Q^{\mathrm{F}}}{Q_{\mathrm{r}}^{\mathrm{F}}}}{\Gamma^{free}} = \frac{\lambda^{\mathrm{F}} \alpha^{\mathrm{F}}}{n \pi d^{\mathrm{F}}} \frac{\left(Q^{\mathrm{F}}\right)^{2}}{Q_{\mathrm{r}}^{\mathrm{F}}} \cos \theta^{\mathrm{F}} \tag{2}$$

COMSOL simulations were used to calculate the enhancement factor of the PC-QD system at both excitation (450 nm) and emission (605 nm) wavelengths.

The energy confinement factors α^{P} and α^{F}, downward radiative quality factor of the pump-resonance mode Q_r^P and total radiative quality factor of the QD fluorescing mode Q_r^F in Eq. (1) and (2) can be obtained through eigenmode simulation. Combining with the experimental spectrum-retrieved total quality factors Q^P and Q^F, we can derive the theoretically predicted enhancement factors.

We performed 3D FEM simulation using COMSOL Multiphysics software with the Radio Frequency Module. The simulation domain contains one PC lattice in the *x*-axis, 100 nm in the *y*-axis and 3 um in the *z*-axis. 0.5 um-thick perfectly matched layers (PML) with scattering boundary conditions are added to both top and the bottom of the structure while periodic boundary conditions (PBC) are applied to *x* and *y* directions of the simulation domain with Bloch wavevector (k_x, k_y). For all the dielectrics: water, SiO_2 and Si_3N_4, we assume they are weakly dispersive and use constant refractive indices $n_{water} = 1.33$, $n_{SiO_2} = 1.47$ and $n_{Si_3N_4} = 2.04$. The total radiative quality factor can be directly obtained by the eigenfrequency solver. We further can obtain the time-averaged radiation energy flow $|\boldsymbol{S}| = \sqrt{emw.Poavx^2 + emw.Poavy^2 + emw.Poavz^2}$ along the *z*-axis in the upper half-plane or lower half-space, and compute the average to obtain the $S^{\mathrm{P,down(up)}}$ and $S^{\mathrm{P,tot}} = S^{\mathrm{P,down}} + S^{\mathrm{P,up}}$. The downward radiative quality factor of the pump-resonance mode

can be derived as $Q_r^P = Q_{r,tot}^{\mathrm{P}} \cdot \frac{S^{P,tot}}{S^{P,down}}$. As the electric field can be expressed as $\boldsymbol{E}(x,y,z) = \boldsymbol{E}(x,z)e^{ik_y y}$, the 3D integral can be reduced to a 2D integral as $\int_\Omega \varepsilon_r(x,y,z)\left|\boldsymbol{E}\right|^2 dxdydz = \int_\Omega \varepsilon_r(x,z)\left|\boldsymbol{E}(x,z)\right|^2 dxdydz = L\int_\sigma \varepsilon_r(x,z)\left|\boldsymbol{E}(x,z)\right|^2 dxdz$. Thus, the energy confinement factors can be calculated on any xOy plane with $\alpha = \frac{\int_{QD\,layer} \varepsilon_r(x,z)\left|\boldsymbol{E}(x,z)\right|^2 dxdz}{\int_{Whole\,space} \varepsilon_r(x,z)\left|\boldsymbol{E}(x,z)\right|^2 dxdz}$, or ($\alpha^{\mathrm{P}} = \int_{QD\,layer}\left|\boldsymbol{E}_{\boldsymbol{k},\omega_k}^{\mathrm{P}}(\boldsymbol{r})\right|^2 d\boldsymbol{r}$ while $E_{k,\omega_k}^P(r)$ is the normalized) where the QD layer is a 4-nm thick layer above the PC grating surface. We should note that we are not performing any integral in PML.

5. Enhance spontaneous emission (Purcell factor calculation)

Under the weak coupling regime, the modification of the LDOS by the dyadic Green's function for a given environment can be evaluated by the classical Poynting's theorem. As a result, the rate of spontaneous emission is directly proportional to the power emitted by a dipole source in the environment of interest [1]. Therefore, the Purcell factor can be calculated by measuring the ratio of the emitted dipole radiation power with and without the presence of the PC at different wavelengths. The numerical simulations were performed using finite difference time domain software (Lumerical FDTD). A monochromatic dipole source was placed 1 nm above the center of the PC surface and surrounded by a 3D box of monitors to record the power emitted from all directions. The simulated PC consisted of 215 periods enclosed by perfectly matched layers in all directions. Based on the structural symmetries of the PC, additional symmetric boundary conditions were used to reduce the simulation time. The mesh was created by using 14 mesh elements per wavelength with a finer mesh step size of 1 nm at the PC-dipole interface. The 2D

Purcell enhancement map was created by placing the dipole source throughout the surface of the grating structure.

6. Collection Efficiency (CE) calculation

The far field radiation pattern was calculated by collecting the fields just above and below the dipole source on the PC/glass and further projecting the near field to far field radiation. The Collection Efficiency was calculated using the Eq. 3.

$$CE = 2\pi \int_{0}^{\theta_{NA}} \left(\frac{d^2S}{d\Omega^2}\right) sin\theta d\theta \tag{3}$$

where the θ is the angle of emission, θ_{NA} is largest collection angle given by $\theta_{NA} = sin^{-1}(NA)$, and $d^2S/d\Omega^2$ is the emission per unit solid angle. Furthermore, the CE calculation also included the losses encountered due to the substrate-air interface by applying the Fresnel coefficients.

7. Back Focal Plane (BFP) Image Simulation.

The 2D far field projection also corresponds well with the experimental BFP image since both are a Fourier transform of the QD's signal in the spatial domain. In order to simulate the spectra distribution of QD-605 emission we have used a series of monochromatic point sources which intensity in the spatial frequency domain $I(k_x, k_y, \lambda)$ was averaged over the experimentally obtained emission density per unit wavelength ($\sigma(\lambda)$) given by Eq. 4.

$$< \mathrm{I}(\mathrm{k_x}, \mathrm{k_y}) > \; = \int_{\lambda_{min}}^{\lambda_{max}} \sigma(\lambda) \mathrm{I}(\mathrm{k_x}, \mathrm{k_y}, \lambda) \mathrm{d}\lambda \tag{4}$$

8. Optical Setup for Time-Resolved Photoluminescence (TRPL) Measurement

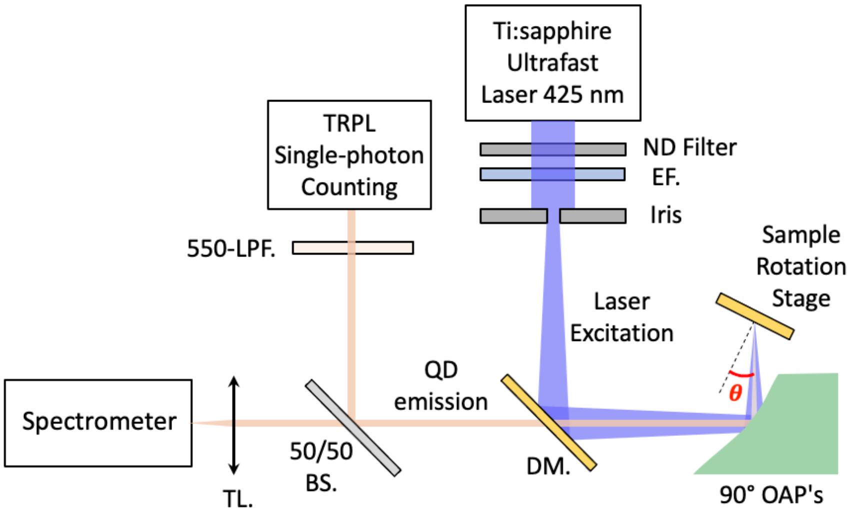

Supplementary Figure S4. Schematic of the Time-Resolved Photoluminescence (TRPL) Experimental Setup. EF: emission filter, DM: dramatic Mirror, OAPs: Off-Axis Parabolic. TL: tube lens, LPF: Long-pass Filter (emission filter). $\theta = 26°$.

9. Quantum Yield (QY) measurements

For fluorescence QY measurements, a dilute QD sample (absorbance ~0.02 at 491 nm) was prepared. The absorption and emission spectrums were acquired as Fig. S5. The same set of spectra were acquired for a reference (fluorescein in 0.1M NaOH, QY.92%). The relative QY was calculated using the following equation.

$$\frac{\phi_{f,QD}(\lambda_{ex,QD})}{\phi_{f,Ref}(\lambda_{ex,Ref})} = \frac{\dfrac{FL_{QD}(\lambda_{ex,QD})}{A_{f,QD}(\lambda_{ex,QD})}}{\dfrac{FL_{Ref}(\lambda_{ex,Ref})}{A_{f,Ref}(\lambda_{ex,Ref})}} \frac{n_{QD}^2}{n_{Ref}^2} \quad (5)$$

where Φ_f, is the fluorescence QY, FL is the total fluorescence intensity (the integrated area of the emission spectrum in wavelength scale) with excitation at λ_{ex} normalized by the intensity of the excitation light, A_f is the absorption factor (or absorptance) at λ_{ex} and n is the refractive index of

the solvent. A_f is the fraction of incident light that is absorbed by the sample which is expressed as:

$$A_f = 1 - T = 1 - 10^{-A} \quad (6)$$

where T and A are the transmittance and the absorbance, respectively. A_f is proportional to the number of photons absorbed by the sample.

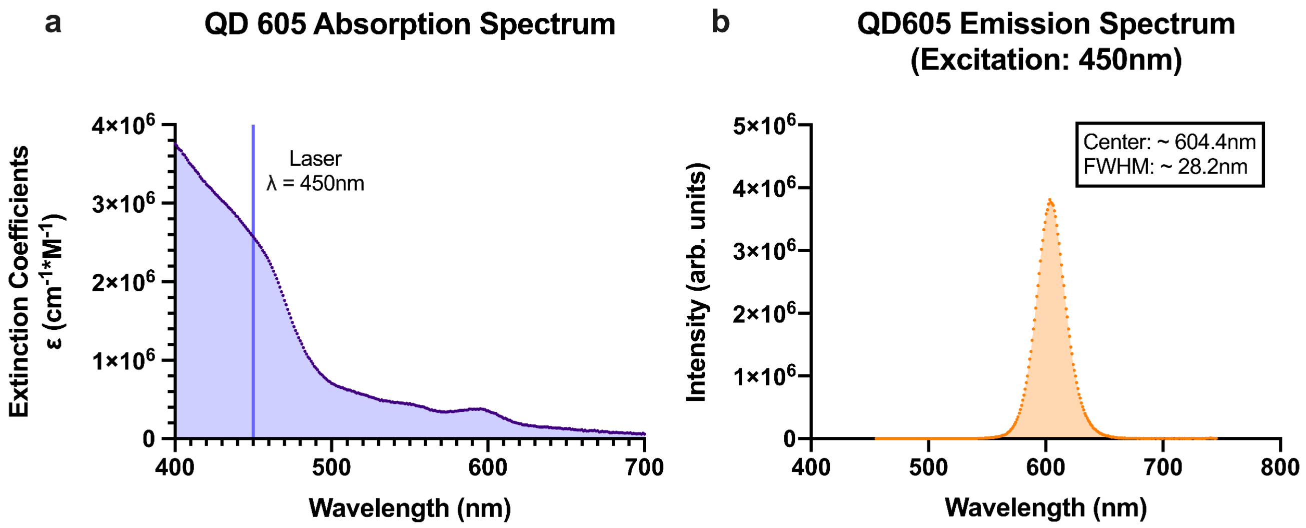

Supplementary Figure S5. Quantum dots (QDs) Optical Properties Calibration. (a) QD-605 absorption spectrum and (b) emission spectrum when excitation using laser 450nm. QD emission centered at 604.4nm with a FWHM of ~28nm.

10. QDs hydrodynamic diameter characterization by DLS

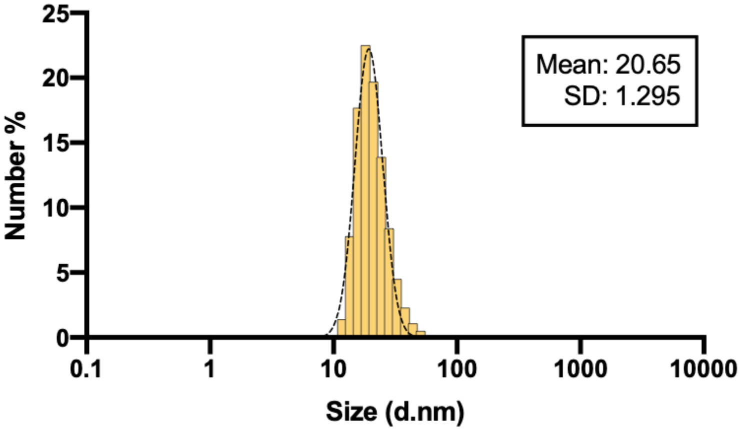

Supplementary Figure S6. Hydrodynamic size distribution of QD605. The average hydrodynamic diameter measured by DLS is 20.65 nm with a standard deviation of 1.295nm.

11. QDs characterization by TEM imaging

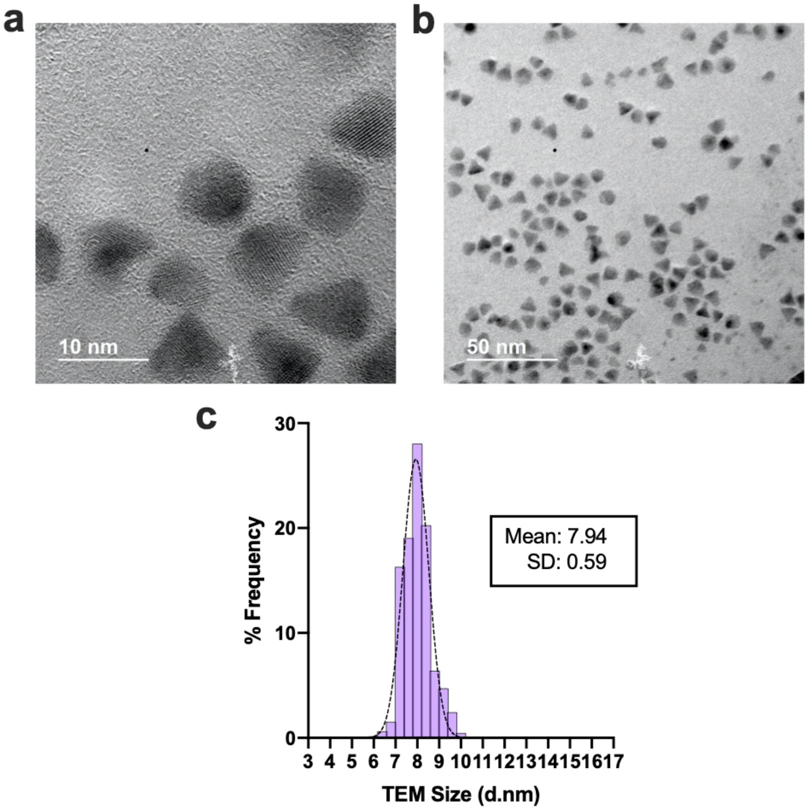

Supplementary Figure S7. QD605 particle hard-size distribution analysis by TEM imaging. Representative TEM images for commercially purchased QDs at (a) 500K and (b) 100K

magnifications. (c) Practical size distribution analysis of QD605. The average diameter of QD605 with complete core–shell structure is 7.94 ± 0.59 nm (number of analyzed QDs > 400).

12. Total enhancement factor calculations

For theoretical total enhancement factor:

Assuming a 4-nm thick QD layer above the PC grating surface, we have the theoretical $\alpha^{\mathrm{P}} = 0.86\%$, $\alpha^{\mathrm{F}} = 0.98\%$ from COMSOL simulation.

Then,

$$\begin{aligned}\Lambda_{total,sim} &= \Lambda_{excitation} \cdot \Lambda_{extraction} \cdot \eta_{QE} \cdot C.E. \\ &= \frac{2\lambda_{laser}}{\pi nd} \frac{\alpha^{P}\left(Q^{P}\right)^{2}}{Q_{\mathrm{r}}^{P}} \cdot \frac{\lambda^{\mathrm{F}}\alpha^{\mathrm{F}}}{n\pi d^{\mathrm{F}}} \frac{\left(Q^{\mathrm{F}}\right)^{2}}{Q_{\mathrm{r}}^{\mathrm{F}}} \cos\theta^{\mathrm{F}} \cdot \frac{QE_{pc}}{QE_{i}} \cdot C.E. \\ &\approx 21.84 \times 34.39 \times 1.67 \times 3.5 \approx 4.390 \times 10^{3}\end{aligned} \tag{7}$$

For experimentally resolved total enhancement factor:

$$\begin{aligned}\Lambda_{total,\exp} &= \Lambda_{excitation,\exp} \cdot (\Lambda_{extraction} \cdot \eta_{QE})_{\exp} \cdot C.E. \\ &\approx 23.14 \times 38.63 \times 3.5 \approx 3.129 \times 10^{3}\end{aligned} \tag{8}$$

13. Z-distance dependence of total enhancement factor

We investigated the Z-distance dependent total enhancement factor with the same simulation model but run a series of test by parameter sweeping in the QD Z-height as a variable d. The simulated enhancement factor Λ (normalized to the 4-nm result) is shown in the Fig. S8 (b). As expected, the greater the distance between QDs and the PC surface, the smaller the electric field experienced by the QD, which leads to a z-dependent enhancement factor. The plot shows that QD

enhancement factor decreases approximately linearly as the QD is moving away from the PC surface for the first ~20 nm in z-height, and that within our anticipated region of QD displacement of 0-10 nm for our miRNA assay, the QDs will be between 100-88% of their maximal enhancement. When QDs are more than 60nm away from the surface, there will be almost no enhancement effects.

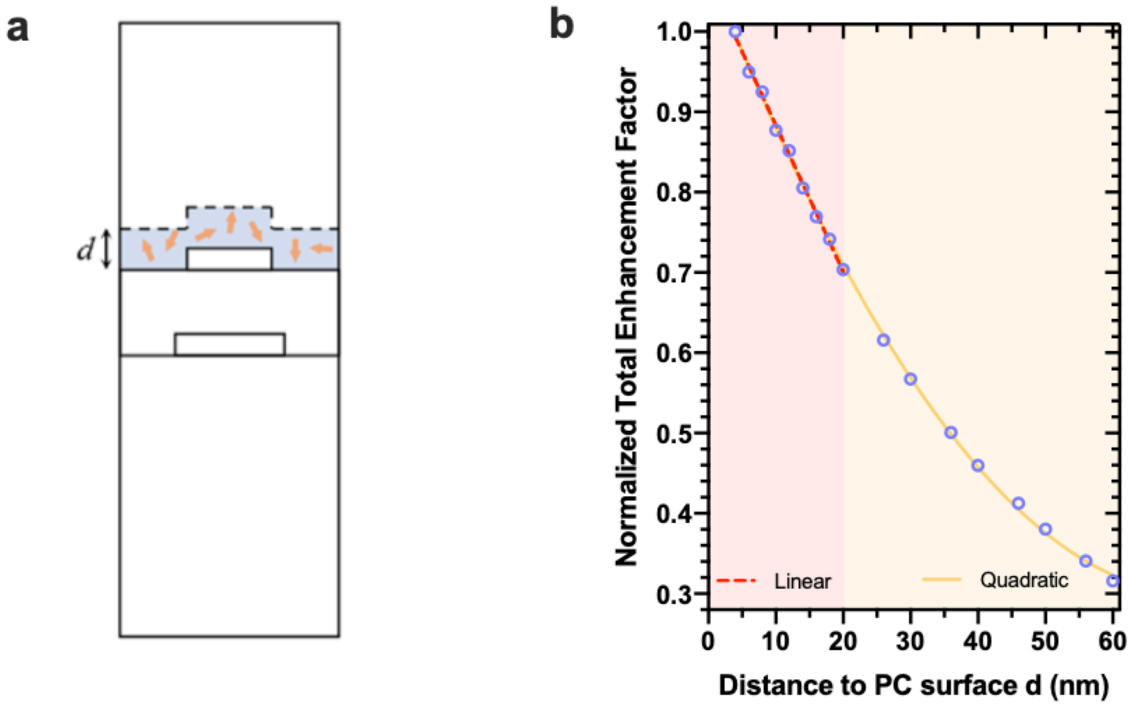

Supplementary Figure S8. Z-distance dependence of total enhancement factor. (a) Schematic illustration of QD layer and the integration region above PC. (b) Simulated distance dependence of the enhancement factor with fitting. A linear model: y = a + bx was used to fit when the distance smaller than 20nm, where y is the normalized total enhancement and x is the distance above PC surface. The linear fitting equation was $y =$ 1.066+ -0.0183x with R^2= 0.997 (the red dash line). A quadratic model: $y =$ 1.079+ -0.0214x+0.00015 x^2 was used to fit when d between 4 to 60nm with R^2= 0.999 (the yellow line).

We here provide a simple but accurate explanation to the linear decrease with small *d*. We take the excitation enhancement factor as an example. $\Lambda_{excitation}$ is proportional to α^P / d, i.e.,

electromagnetic energy of pump-mode stored in the QD layer and the evanescent electric field can be written as $\sum_{\boldsymbol{G}} E_{\boldsymbol{G}}^{P} e^{-z/z_{\boldsymbol{G}}^{P}} e^{i(\boldsymbol{k}_{\parallel}+\boldsymbol{G})\cdot \boldsymbol{r}}$, by using the Bloch theorem. Here $k_{\parallel}$ is the in-plane wavevector, $\boldsymbol{G}$ is any of the reciprocal vectors and $z_{\boldsymbol{G}}^{P} = 1/\sqrt{(\omega/nc)^2 + |\boldsymbol{k}_{\parallel} + \boldsymbol{G}|^2}$ is the corresponding evanescent tail length. Thus, we have

$$\Lambda_{excitation} \propto \alpha^{P} / d \propto \int_{QD\_layer} \left| \sum_{\boldsymbol{G}} E_{\boldsymbol{G}}^{P} e^{-z/z_{\boldsymbol{G}}^{P}} e^{i(\boldsymbol{k}_{\parallel}+\boldsymbol{G})\cdot \boldsymbol{r}} \right|^2 dxdydz / d$$

$$\propto \sum_{\boldsymbol{G}} \left| E_{\boldsymbol{G}}^{P} \right|^2 \int_0^d e^{-2z/z_{\boldsymbol{G}}^{P}} dz = \sum_{\boldsymbol{G}} \left| E_{\boldsymbol{G}}^{P} \right|^2 \frac{z_{\boldsymbol{G}}^{P}}{2d} (1 - e^{-2d/z_{\boldsymbol{G}}^{P}}) \sim \sum_{G} \left| E_{\boldsymbol{G}}^{P} \right|^2 (1 - (d / z_{\boldsymbol{G}}^{P})) = 1 - \frac{\sum_{\boldsymbol{G}} \left| E_{\boldsymbol{G}}^{P} \right|^2 / z_{\boldsymbol{G}}^{P}}{\sum_{\boldsymbol{G}} \left| E_{\boldsymbol{G}}^{P} \right|^2} d,$$

which is linear with small d, and same for extraction enhancement factor. The total enhancement can be determined as

$$\Lambda \propto \Lambda_{excitation} \Lambda_{extraction} \sim (1 - \frac{\sum_{\boldsymbol{G}} \left| E_{\boldsymbol{G}}^{P} \right|^2 / z_{\boldsymbol{G}}^{P}}{\sum_{\boldsymbol{G}} \left| E_{\boldsymbol{G}}^{P} \right|^2} d)(1 - \frac{\sum_{\boldsymbol{G}} \left| E_{\boldsymbol{G}}^{F} \right|^2 / z_{\boldsymbol{G}}^{F}}{\sum_{\boldsymbol{G}} \left| E_{\boldsymbol{G}}^{F} \right|^2} d) \sim 1 - \left[\frac{\sum_{\boldsymbol{G}} \left| E_{\boldsymbol{G}}^{P} \right|^2 / z_{\boldsymbol{G}}^{P}}{\sum_{\boldsymbol{G}} \left| E_{\boldsymbol{G}}^{P} \right|^2} + \frac{\sum_{\boldsymbol{G}} \left| E_{\boldsymbol{G}}^{F} \right|^2 / z_{\boldsymbol{G}}^{F}}{\sum_{\boldsymbol{G}} \left| E_{\boldsymbol{G}}^{F} \right|^2} \right] d,$$

which is linear in d as well. With larger d, the above integration can be expanded to the next order with another quadratic term, as shown in Fig. S8 (b).

14. Purcell factors remain constant for a broad wavelength range

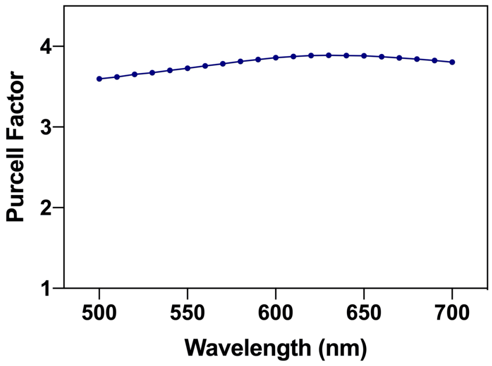

Supplementary Figure S9. Purcell enhancement factor simulation. The simulation results shown the Purcell enhancement factors remain constant for a broad wavelength range from 500nm to 700nm.

15. QD Saturation and the observation of multiexciton generation

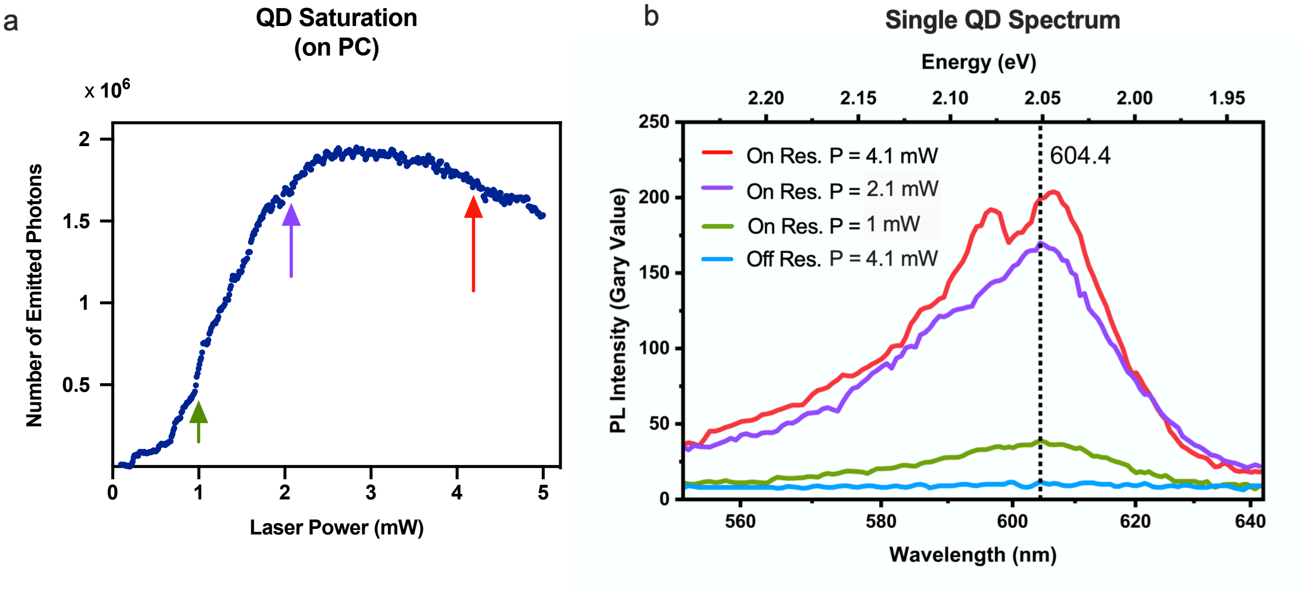

Supplementary Figure S10. QD Saturation. (a) QD saturation happens under PC enhanced condition when input laser power is higher than 3mW. (b) Corresponded spectrums. The red line of QD emission spectrum under laser power = 4.1 mW with on resonance condition. The appearing of side peak at shorter wavelength shown the observation of multiexciton generation. Purple and green line shown the QD spectrums under the on-resonance input laser power of 2.1mW and 1mW respectively. Blue line is spectrum while QD on PC with excitation pump-mode off resonance. All intensity data points are shown the “on state” of single QDs only with continuous-wave excitation under room temperature.

16. Quantum dot conjugation with ssDNA and gel electrophoresis

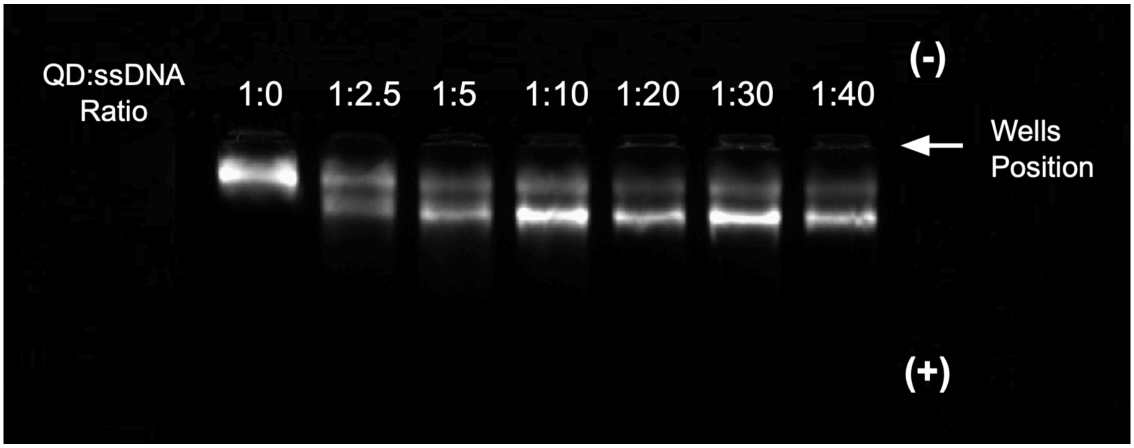

Supplementary Figure S11. Gel electrophoresis image for Quantum dot and ssDNA conjugation.

To demonstrate quantum dot conjugation to DNA, biotin-labeled DNA with 90 bases (Integrated DNA Technologies) was incubated with QD605 streptavidin conjugate (Invitrogen) at 4 °C for 1 hour. Incubation was with different ratios of biotin-DNA. Conjugation was measured using gel electrophoresis in hybrid polyacrylamide-agarose gels (2% polyacrylamide-agarose and 0.5% agarose) at 4 °C. Gel was prepared as previously described [2] by mixing 25 mL of 1% (w/v) melted agarose prepared in water and 25 mL of 4% (v/v) acrylamide prepared in 100 mM of sodium borate buffer. Ammonium persulfate and TEMED were then added to final concentrations of 0.05% (w/v) and 1:2500 (v/v), respectively. Gel was allowed to solidify for 1 hour. Sample was loaded by mixing 10 uL of sample with 2 uL of loading buffer (30 % w/v glycerol in 50 mM sodium borate buffer). Electrophoresis was run at 120 V for 30 min at 4 °C. More than three experiments were repeated independently with similar results. Gel electrophoresis image taken using GeneSys Software.

As shown on Fig. S7, Gel data indicate the conjugation between DNA and streptavidin quantum dots due to the presence of a second band in samples with DNA. A second band with longer migration distance towards the positive side of the gel is seen in quantum dot-DNA conjugates due to the negative charge of the DNA. As expected, increasing ratio of DNA to QDs showed

increasing brightness in the second band compared to the first due to an increase in number of DNA per QD. We choose the ratio of ssDNA: QD as 10:1 since the streptavidin binding sits appear to saturate at higher ratio.

17. Full dose-response curve with model fitting

Based on the r-squared values, our results show that a full range (extended 12-order) of analyte concentrations can be best fitted by a five-parameter logistic model while the middle 9-order of concentrations (10 aM-1 nM) can be well fitted with a log-log "linear" model.

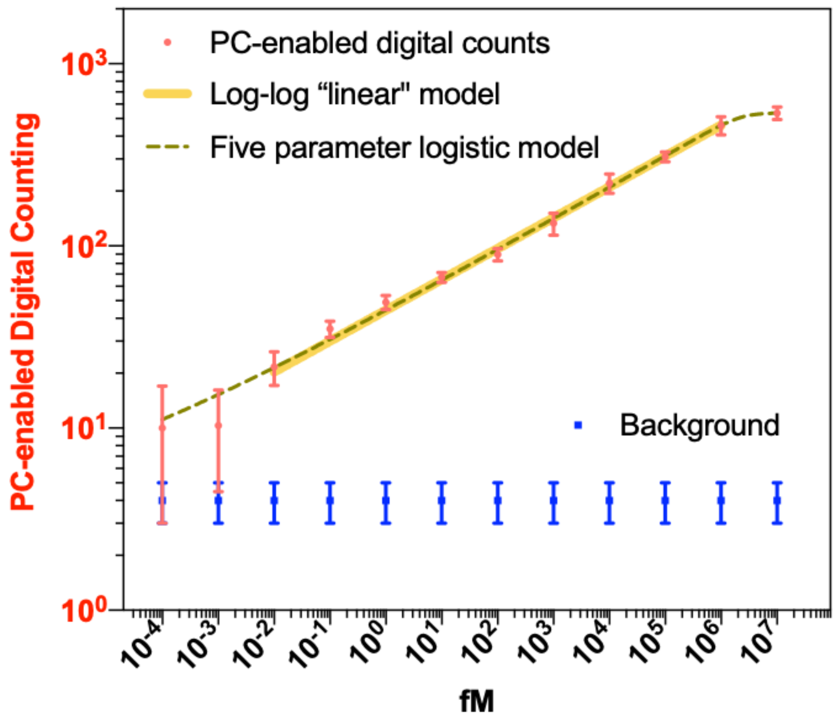

Supplementary Figure S12. Calibration curves of the miR-375 detection assay in human serum. For the full concentration range from 100 zM to10 nM, a five-parameter logistic model: $y = top + \frac{bottom-top}{\left[1+\left(\frac{x}{EC_{50}}\right)^{slope}\right]^{asymmetry}}$ was used to fit the dose response, where x is the concentration of the targeted miRNA molecules, y is the QD counts. The five-parameter logistic fitting equation

was $y = 537.8 + \frac{2.75-537.8}{\left[1+\left(\frac{x}{43297}\right)^{2.162}\right]^{0.081}}$ with R^2= 0.9886 (the green dash line). For the middle concentration range from 10 aM to1 nM, a log-log linear model: log(y) = a*log(x) + b was used to portrait the dose response. The log-log linear regression fitting equation is log(y)=0.1692* Log(x)+1.647 with R^2= 0.9825 (the yellow line). The error bars represent the mean and the standard deviation of three independent assays.

18. Kinetic performance of the sensor

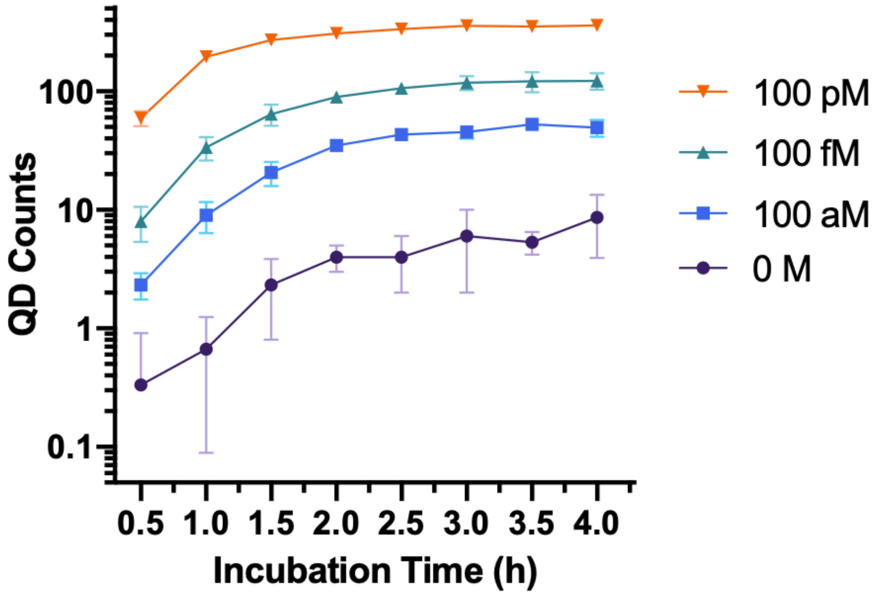

Supplementary Figure S13. The quantification of the detected QDs versus incubation time for four different target concentrations. Each data point represents the average of 3 independent experiments. The error bars represent the mean and the standard deviation of three independent assays. One error bar (at zero) for 0M at 0.5h is not shown since the y-axis is logarithmic in this plot.

19. Conventional analog intensity measurements from glass surface-based assay

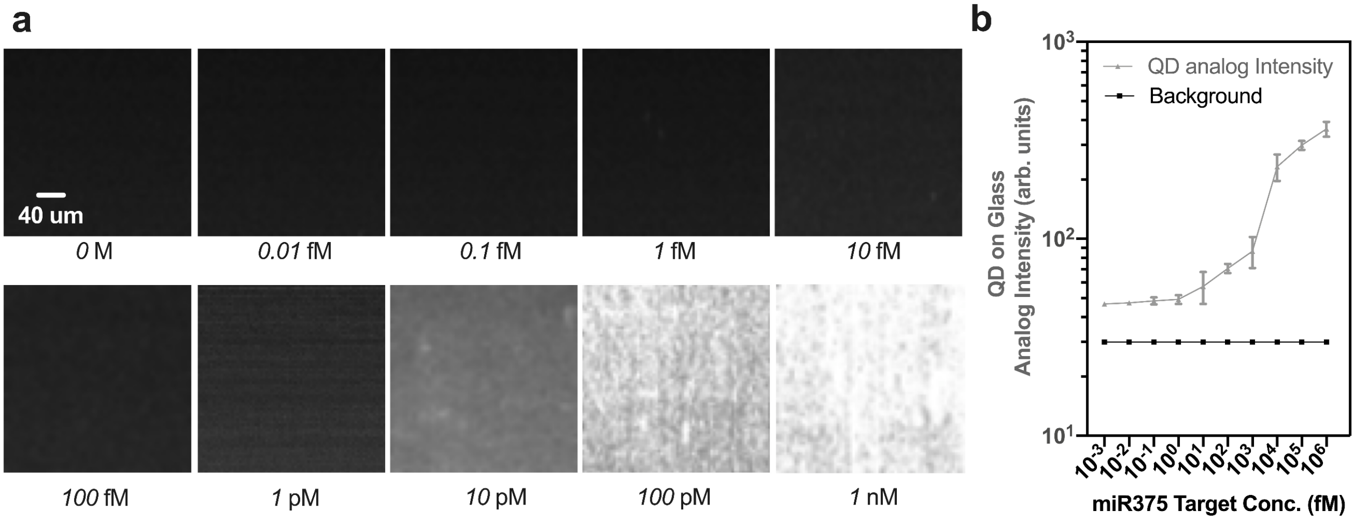

Supplementary Figure S14. Conventional unenhanced analog ensemble intensity results. Line scanning images for performing the same assay on a glass substrate. miRNA target concentration: 10 aM to 1 nM. FOV: 300 µm *300 µm. Scale bar: 40 µm. Data are averages from more than 9 FOVs, and error bars indicate the mean and the standard deviation between 3 independent replicas.

20. Dynamic characteristics of QD-trajectories Single-base mismatch discrimination test

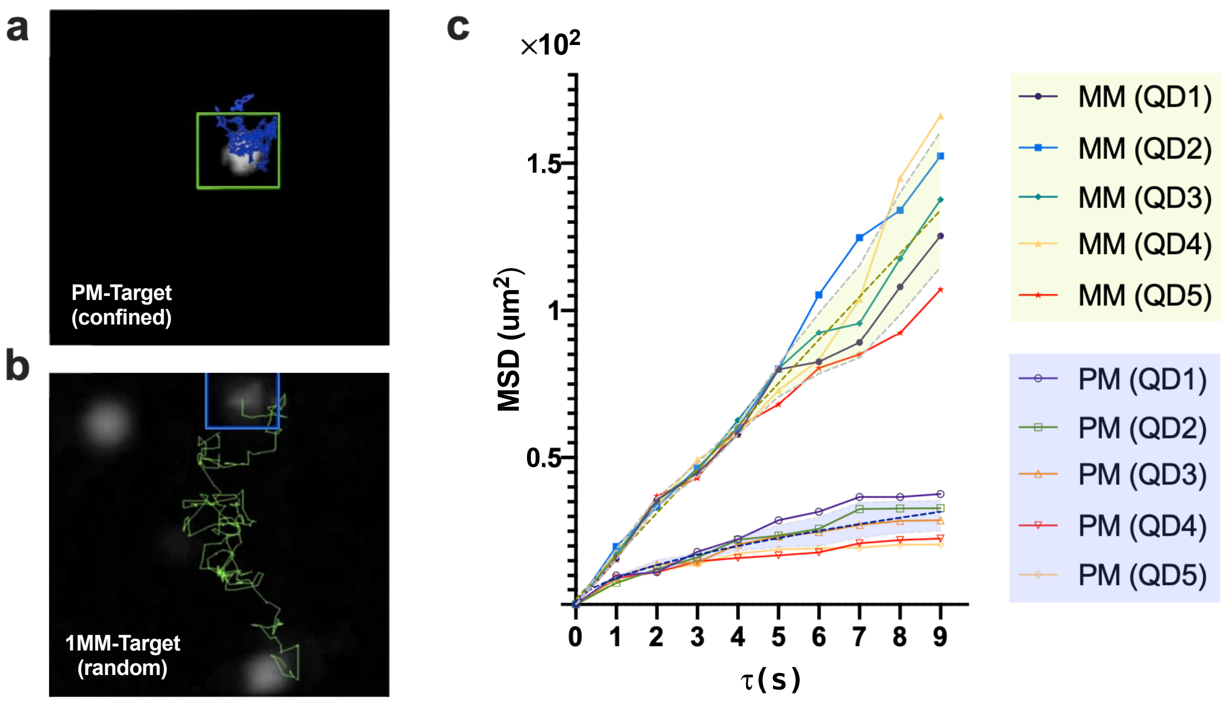

Supplementary Figure S15. Single-base mismatch discrimination using QD-trajectories. Trajectories with 225 time-steps (9s total) for both for (a) perfect match (PM) and (b) single-base mismatch (1MM) groups. PM trajectories show a "confined diffusion" pattern (blue) while 1MM groups appear a "random walk" pattern (green). (c) MSD plot respect to τ, the shade region are average values (green dash line: PM; blue dash line: 1MM) pulse with error bands for five trajectories from each testing group.

21. PC biosensor system

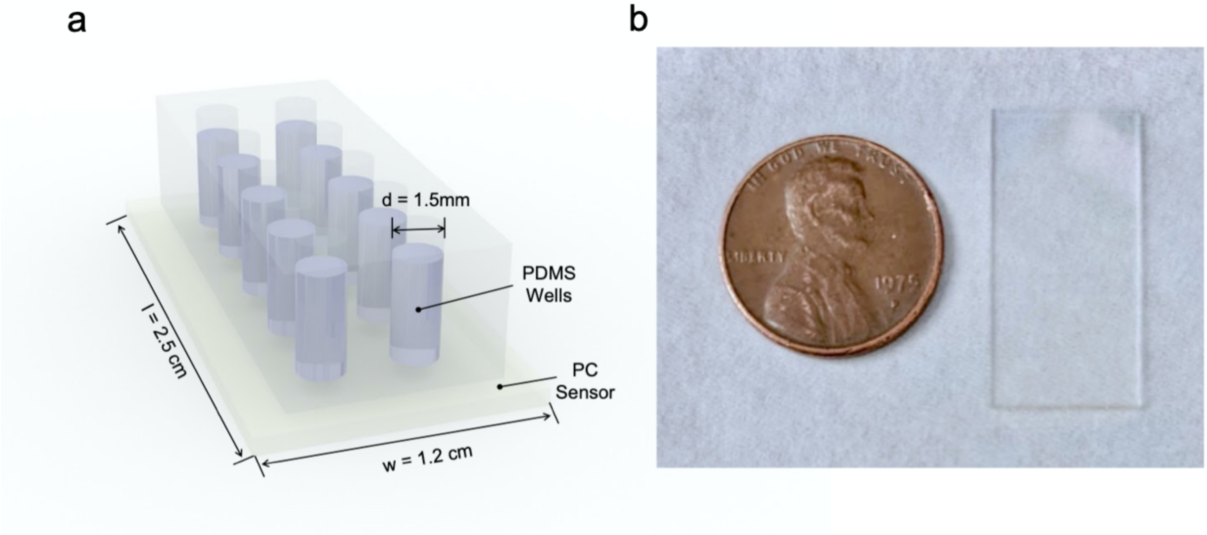

Supplementary Figure S16. PC biosensor system. (a)Schematic drawing of the PC biosensor with applied array of PDMS wells. Well diameter: 1.5 mm; Sample volume: 45 mL. (b)A photo of the actual diced PC next to a coin. Dimensions: 12 mm×25 mm×0.8 mm.

22. Nucleic acids

The oligonucleotides (oligos) used in this study were purchased from Integrated DNA Technologies (IDTDNA Inc, Coralville, Iowa). The probe oligo is functionalized with a biotin at the 3'-end, followed by high-performance liquid chromatography (HPLC) purification. The PC capture probe oligo carries a 5'-primary amine for immobilization to PC surface. Sequences of all the relevant oligos are shown as follows:

PC capture:	5'- NH_2-TTTTTTCACGCGAGC-3'
Probe:	5'-CGAACGAACAAATTTTT-Biotin-3'
Target miR-375 (PM):	5'-UUUGUUCGUUCGGCUCGCGUGA-3
4st nucleotide mutation ($1MM_4$):	5'-UUUAUUCGUUCGGCUCGCGUGA-3

6st nucleotide mutation (1MM$_6$): 5'-UUUGUCCGUUCGGCUCGCGUGA-3

8st nucleotide mutation (1MM$_8$): 5'-UUUGUUCCUUCGGCUCGCGUGA-3

23. Mismatch Index NUPACK Simulation

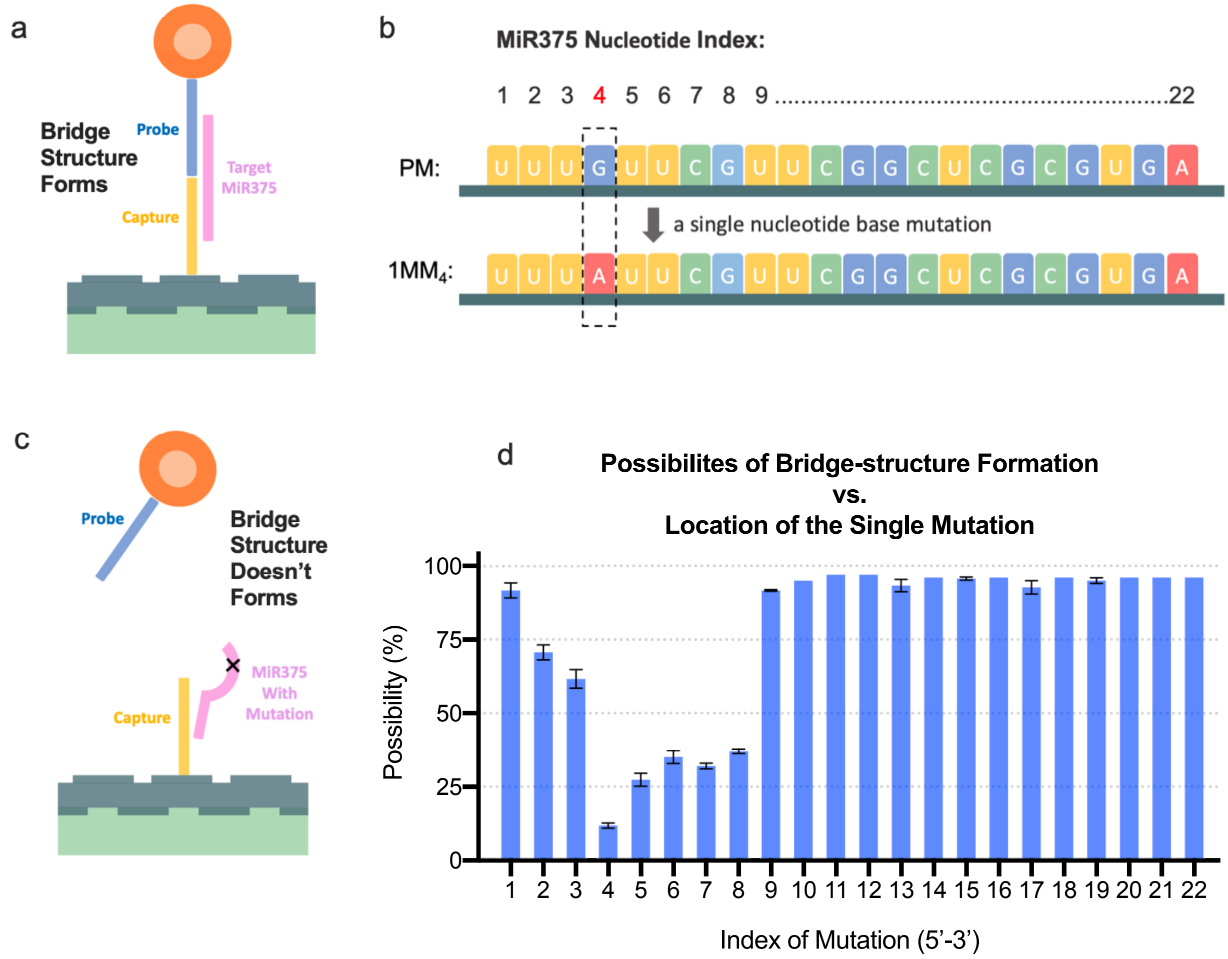

Supplementary Figure S17. (a). Illustration of Bridge structure formation and active counting with perfect-match (PM) target. (b). Example of a single nucleotide base mutation of miR375 at index #4 (1MM$_4$), where "G" mutates to "A". (c) Illustration where Bridge structure formation doesn't form well with a single base mutation. QD-probe are not pulled down to the enhanced region without bridge. (d). NUPACK simulation for the average possibilities of bridge-structure

formation vs. mismatch index. One index-mutation can have three different forms (eg. G to A/U/C) The error bar shows the standard deviations of three different mutation forms in the same index.

References

1 Novotny, L. & Hecht, B. *Principles of nano-optics*. 2nd edn, (Cambridge University Press, 2012).

2 Le, P., Chitoor, S., Tu, C., Lim, S. J. & Smith, A. M. Compact Quantum Dots for Quantitative Cytology. *Methods Mol Biol* **2064**, 147-158, doi:10.1007/978-1-4939-9831-9_12 (2020).
