# Supplementary material for: Photonic crystal enhanced fluorescence emission and blinking suppression for single quantum dot digital resolution biosensing
Source: Nat Commun. 2022 Aug 8;13:4647. doi: 10.1038/s41467-022-32387-w (PMC9360002; doi:10.1038/s41467-022-32387-w)
Supplement: Supplementary file 3 — Reporting Summary [file 41467_2022_32387_MOESM3_ESM.pdf]

nature portfolio

Corresponding author(s): Brian T. Cunningham

Last updated by author(s): 07/22/2022

Reporting Summary

Nature Portfolio wishes to improve the reproducibility of the work that we publish. This form provides structure for consistency and transparency in reporting. For further information on Nature Portfolio policies, see our Editorial Policies and the Editorial Policy Checklist.

Statistics

For all statistical analyses, confirm that the following items are present in the figure legend, table legend, main text, or Methods section.

n/a	Confirmed	
[ ]	[x]	The exact sample size (n) for each experimental group/condition, given as a discrete number and unit of measurement
[x]	[ ]	A statement on whether measurements were taken from distinct samples or whether the same sample was measured repeatedly
[ ]	[x]	The statistical test(s) used AND whether they are one- or two-sided<br>*Only common tests should be described solely by name; describe more complex techniques in the Methods section.*
[x]	[ ]	A description of all covariates tested
[x]	[ ]	A description of any assumptions or corrections, such as tests of normality and adjustment for multiple comparisons
[ ]	[x]	A full description of the statistical parameters including central tendency (e.g. means) or other basic estimates (e.g. regression coefficient) AND variation (e.g. standard deviation) or associated estimates of uncertainty (e.g. confidence intervals)
[ ]	[x]	For null hypothesis testing, the test statistic (e.g. F, t, r) with confidence intervals, effect sizes, degrees of freedom and P value noted<br>*Give P values as exact values whenever suitable.*
[x]	[ ]	For Bayesian analysis, information on the choice of priors and Markov chain Monte Carlo settings
[x]	[ ]	For hierarchical and complex designs, identification of the appropriate level for tests and full reporting of outcomes
[x]	[ ]	Estimates of effect sizes (e.g. Cohen's d, Pearson's r), indicating how they were calculated

Our web collection on statistics for biologists contains articles on many of the points above.

Software and code

Policy information about availability of computer code

Data collection	The fluorescent image acquisitions were performed by Hamamatsu HCImage software; The numerical simulations were performed using finite difference time domain software (Lumerical FDTD) and COMSOL Multiphysics software; The cross-sectional height profiles of the PC were collected with Cypher AFM using Asylum Research SPM. TEM images of the QDs were collected using the JEOL 2100 Cryo TEM operates at 200kV. SEM images of the PC-QD sample were obtained using a Hitachi S-4800 field emission SEM. DLS data were obtained from Malvern Zetasizer to measure QD diameters. The gel image was collected by GeneSys Software for G:BOX F3 (Syngene); The Time-Resolved Photoluminescence data of QD lifetime was measured by SPCImage NG Data Analysis Software with the bh TCSPC Package. The QD spectrum data were measured by HORIBA UV-VIS Spectroscopy.
Data analysis	The fluorescent images were analyzed by Hamamatsu HCImage software (Ver. 4.6.1.19) and ImageJ-Fiji (Ver. 2.0.0). The numerical simulations were analyzed using finite difference time domain software (Lumerical FDTD, Ver. 2020a) and COMSOL Multiphysics software (Ver. 5.6); The bridge-assay design was analyzed using Nucleic Acid Package (NUPACK, Ver. 4.0) web server; The AFM data were analyzed with Asylum Research SPM and Gwyddion Software (Ver. 2.59). TEM and SEM images were analyzed using ImageJ-Fiji (Ver. 2.0.0). DLS data were analyzed by Zetasizer software (Malvern, Ver. 7.10). Gel image was analyzed by GeneTools Software for G: BOX F3 (Syngene, Ver. 1.8.5); The Time-Resolved Photoluminescence data of QD lifetime was analyzed by SPCImage NG Data Analysis Software with the bh TCSPC Package (Ver. 8.3). QD spectrum data were analyzed using HORIBA Scientific SynerJY software (Ver. 3.5) . GraphPad Prism (Ver. 9.0.1) was used to make the dose-repose plots for miR sensing and the associated statistical analyses of data; Adobe Illustrator (Ver. 25.2.3) was used to generate figures in the paper; MATLAB (Ver. R2018a) was using for QD images and video analysis.

For manuscripts utilizing custom algorithms or software that are central to the research but not yet described in published literature, software must be made available to editors and reviewers. We strongly encourage code deposition in a community repository (e.g. GitHub). See the Nature Portfolio guidelines for submitting code & software for further information.

Data

Policy information about availability of data

All manuscripts must include a data availability statement. This statement should provide the following information, where applicable:
- Accession codes, unique identifiers, or web links for publicly available datasets
- A description of any restrictions on data availability
- For clinical datasets or third party data, please ensure that the statement adheres to our policy

The data that support the findings of this study are available within the article and its Supplementary Information, or from the corresponding author upon request.

Field-specific reporting

Please select the one below that is the best fit for your research. If you are not sure, read the appropriate sections before making your selection.

- [x] Life sciences
- [ ] Behavioural & social sciences
- [ ] Ecological, evolutionary & environmental sciences

For a reference copy of the document with all sections, see nature.com/documents/nr-reporting-summary-flat.pdf

Life sciences study design

All studies must disclose on these points even when the disclosure is negative.

Sample size	For all miR375 assays, triplicate assays were used for three independent tests. The experimental enhancement factors calculate based on a sample size of 100 individual QDs. The TEM QD size analysis calculate based on more than 400 individual QDs. Three biological triplicates are often used in the bio-assay field. Sample sizes above 50 are often used in particle characterizations.
Data exclusions	No data were excluded. All data obtained in analyses are presented.
Replication	miR375 assays were performed in biological triplicate every 1-2 weeks. All attempts at replication were successful.
Randomization	The allocation for fluorescent image, SEM, TEM, AFM, and DLS used in this study were randomized by taking imaging at random locations and collecting data at a random time.
Blinding	Four of our analytical methods (SEM, TEM, AFM, and DLS) used in this study were characterization measurements and, unlike biological or in vivo assays, do not routinely use blinded samples since the influence of the person who performs the experiments is negligible. The miR375 direct counting assay was blinded as the samples were labeled, prepared, and analyzed by three different people.

Reporting for specific materials, systems and methods

We require information from authors about some types of materials, experimental systems and methods used in many studies. Here, indicate whether each material, system or method listed is relevant to your study. If you are not sure if a list item applies to your research, read the appropriate section before selecting a response.

Materials & experimental systems

n/a	Involved in the study	
x		Antibodies
x		Eukaryotic cell lines
x		Palaeontology and archaeology
x		Animals and other organisms
x		Human research participants
x		Clinical data
x		Dual use research of concern

Methods

n/a	Involved in the study	
x		ChIP-seq
x		Flow cytometry
x		MRI-based neuroimaging
